# Meta-analysis confirms BCL2 is an independent prognostic marker in breast cancer

**DOI:** 10.1186/1471-2407-8-153

**Published:** 2008-05-29

**Authors:** Grace M Callagy, Mark J Webber, Paul DP Pharoah, Carlos Caldas

**Affiliations:** 1 Department of Pathology, National University of Ireland, Galway, Clinical Science Institute, Costello Road, Galway, Ireland; 2 Cancer Research-UK Department of Oncology, Cancer Research-UK Genetic Epidemiology Unit and EPIC, Strangeways Research Laboratory, Worts Causeway, Cambridge CB1 8RN, UK; 3 Department of Oncology, University of Cambridge and Cancer Research-UK Cambridge Research Institute, Li Ka Shing Centre, Cambridge CB2 0RE, UK

## Abstract

**Background:**

A number of protein markers have been investigated as prognostic adjuncts in breast cancer but their translation into clinical practice has been impeded by a lack of appropriate validation. Recently, we showed that BCL2 protein expression had prognostic power independent of current used standards. Here, we present the results of a meta-analysis of the association between BCL2 expression and both disease free survival (DFS) and overall survival (OS) in female breast cancer.

**Methods:**

Reports published in 1994–2006 were selected for the meta-analysis using a search of PubMed. Studies that investigated the role of BCL2 expression by immunohistochemistry with a sample size greater than 100 were included. Seventeen papers reported the results of 18 different series including 5,892 cases with an average median follow-up of 92.1 months.

**Results:**

Eight studies investigated DFS unadjusted for other variables in 2,285 cases. The relative hazard estimates ranged from 0.85 – 3.03 with a combined random effects estimate of 1.66 (95%CI 1.25 – 2.22). The effect of BCL2 on DFS adjusted for other prognostic factors was reported in 11 studies and the pooled random effects hazard ratio estimate was 1.58 (95%CI 1.29–1.94). OS was investigated unadjusted for other variables in eight studies incorporating 3,910 cases. The hazard estimates ranged from 0.99–4.31 with a pooled estimate of risk of 1.64 (95%CI 1.36–2.0). OS adjusted for other parameters was evaluated in nine series comprising 3,624 cases and the estimates for these studies ranged from 1.10 to 2.49 with a pooled estimate of 1.37 (95%CI 1.19–1.58).

**Conclusion:**

The meta-analysis strongly supports the prognostic role of BCL2 as assessed by immunohistochemistry in breast cancer and shows that this effect is independent of lymph node status, tumour size and tumour grade as well as a range of other biological variables on multi-variate analysis. Large prospective studies are now needed to establish the clinical utility of BCL2 as an independent prognostic marker.

## Background

Breast cancer is a heterogenous disease whose behaviour is determined by the molecular characteristics of the tumour. In clinical practice, we rely on clinico-pathological features to predict tumour behaviour and patient outcome. These are powerful independent prognosticators [1,2] but are imperfect and represent only crude measures of the biological behaviour of a tumour. The power of these factors can be increased when they are used in combination *e.g*. the Nottingham Prognostic Index (NPI) [1] but they cannot predict outcome for all patients [3-5] and, as a result, many patients receive unnecessary treatment.

In the last 30 years, intensive efforts have been made to identify tools to improve prognostication. These range from biological markers to computer-based algorithms such as ADJUVANT! [6] that incorporate clinico-pathological and biological features. Early immunohistochemical studies identified a plethora of biological markers that can be triaged into categories based on the strength of evidence supporting their prognostic role [7]. However, twenty years on only steroid hormone receptors and HER2 are used in clinical practice. The translation of these results has in part been impeded by a lack of robustness of the original research findings. Many studies were small and false positives are the result of a combination of low statistical power and publication bias. Furthermore, many prognostic markers are correlated and small studies have limited power to show independence of effects of novel markers in multivariate analyses.

Prognostic gene-based signatures have been reported with the claim that they out-perform current standards [8,9] although this has been questioned [10-12]. While promising, inconsistencies relating to quality control, tissue handling, sample size and data analysis need to be addressed before the clinical utility of gene-based prognostic signatures can be established. Two prospective clinical trials of prognostic gene signatures are now ongoing [13,14] and, it is hoped, these will provide insight into the utility of widespread gene expression profiling as clinical tools.

A complementary approach uses a limited number of immunohistochemical markers as surrogates for the gene-based signatures. The 'intrinsic subtypes' [15-17] have been reproduced repeatedly in this way and this methodology is likely to be a more realistic approach for routine diagnostic application [18-20]. In order to have clinical application, it is paramount that any marker shows good and poor prognostic groups independently of currently used standards or markers at stringent levels of statistical significance. With this in mind, we recently evaluated a panel of 13 biomarkers by immunohistochemistry (IHC) in a series of over 700 breast cancers and validated the findings in a second consecutive series of 983 cases on a tissue micro-array (TMA) [21]. The panel included proteins whose genes were differentially expressed in the intrinsic subsets defined by microarray profiling [15,16]. We expected that such a panel would have greater prognostic power than any one individual marker but the results showed otherwise: only the anti-apoptotic protein BCL2 was required to predict overall survival independent of traditional parameters in both series. Furthermore BCL2 added to the prognostic power of the NPI.

The BCL2 protein is a member of the bcl family that regulate apoptosis. Its tumorigenic potential has been demonstrated in animal models [22] and is supported by the finding of over-expression of BCL2 in a variety of tumours and in lymphomas in which BCL2 acts as an oncogene [23,24]. In many solid organ tumours, including breast cancer, BCL2 paradoxically appears to exert a tumour suppressor effect, where its expression is associated with favourable prognostic features *e.g*. low grade, oestrogen receptor (ER)-positivity and good outcome [21]. This has been a consistent finding in most reports but the association with outcome was limited to univariate analysis in many.

The aim of this study was to evaluate the prognostic role of BCL2 in breast cancer by sytematically reviewing the available evidence. To this end, we identified all published reports that assessed the relationship between BCL2 and outcome in breast cancer and performed a meta-analysis using standard statistical techniques.

## Methods

Reports investigating the prognostic role of BCL2 in breast cancer were selected for review using a search of PubMed from 1994–2006 using the following criteria: '(bcl-2 and breast) AND (prognosis* [Title/Abstract] OR (first [Title/Abstract] AND episode [Title/Abstract]) OR cohort [Title/Abstract])' and also '(bcl-2 and breast) AND (incidence [MeSH:noexp] OR mortality [MeSH Terms] OR follow up studies [MeSH:noexp] OR prognos* [Title/Abstract] OR predict* [Title/Abstract] OR course* [Text Word])' limited to the English language. Three hundred and thirty three citations were retrieved. Eligible reports were those that examined the association between expression of BCL2 by IHC and either overall survival (OS) and/or disease free survival (DFS) in a clinical series of invasive breast cancer. Reports considered ineligible for the meta-analysis were reviews; those of *in situ *carcinoma or precursor lesions or of only male breast cancer; those that used cell lines or techniques other than IHC; and reports where either OS or DFS were not used as clinical endpoints; or where the association between another marker and outcome was being examined and data for BCL2 was not presented. Authors that published multiple reports on a single series were included once. The bibliography of the reports was also searched by hand.

Fifty-three eligible studies were identified. Small studies are more prone to publication bias and failure to report a hazard ratio (HR) can be taken as an indicator of study quality [25]. We therefore included the seventeen studies that were based on at least 100 patients and had also reported an estimate of the HR [21,26-41]. The remaining 36 reports (Additional files 1 and 2) included 11 reports that were either based on a small number of cases (≤ 100) or were larger studies that did not report a HR but did provide sufficient data to estimate the HR according to the method of Parmer *et. al*. [42]. These were analysed separately to determine if these studies would introduce significant bias [43-53]. The remaining 25 studies, including nine using small series, provided insufficient data for analyses and were excluded (Additional files 1 and 2).

### Design of the meta-analysis

Pooled estimates of the HRs were obtained using both fixed-effect and random-effects meta-analysis using the inverse-variance weighting method based on published confidence intervals for the HRs [54]. For those studies that did not report the HR but did provide sufficient information on survival by BCL2 status the we estimated the HR and confidence intervals according to the method of Parmer *et al*. [42].

In one report [27], the upper confidence limit for the univariate HR was clearly incorrect and inconsistent with the lower confidence limit. The variance for this HR was estimated based on the lower confidence limit only. Statistical heterogeneity between studies was assessed using the among-study variance (s2) and the statistic I2 [55]. A funnel plot and Egger's regression test for funnel plot asymmetry [56] were used to look for the presence of a small-study effect that might be due to publication bias.

In many studies, in addition to univariate analysis, the risks were adjusted for other prognostic factors. We performed separate analyses based on adjusted and unadjusted HRs for both DFS and OS. For the purposes of the analysis we converted all hazard ratios to a comparison of BCL2 negative tumours with BCL2 positive tumours.

All analyses were carried out using Stata version 9 (Stata Corporation, College Station, TX, USA).

## Results

Seventeen papers reporting 18 large case series with complete statistical data were included in the main analyses. These evaluated the role of BCL2 as a predictor of OS or DFS in 5,892 cases of breast cancer (Additional files 3 and 4). Median follow-up ranged from 0.2 to 472 months (average 92.1 months). In total, 2,619 cases of node-negative disease and 3,963 cases of node-positive disease were analysed. In 333 cases, nodal status was either unknown or not indicated. Five of the 18 series comprised consecutive cases unselected for specific characteristics [21,29,31,33,39]. The remainder consisted of cases that were either accrued onto clinical trials [21,32,35,37] or selected according to defined eligibility criteria *i.e*. node-negative [30,41] or node-positive [26,28,34,36,38,40] disease, or tumour size [27]. Unfortunately, the number of studies was too small for formal meta-analysis for each of these end-points.

Fourteen studies reported on the effect of BCL2 on DFS of which three reported only unadjusted [32,34,40] and six reported only multivariate adjusted [29,30,33,36,37,39] hazards and five reported both [26,27,35,38,41]. Where multivariate analysis was performed the parameters included varied. Clinico-pathological variables were incorporated in most analyses with the following exceptions. Tumour size was excluded in two reports [35,37]. Nodal status was omitted in five studies [26,30,35,37,41] although these were selected series that consisted entirely of either node positive [26], node negative [30,41] or metastatic disease [35,37]. Tumour grade was also excluded in five reports [26-28,37,38]. The other biomarkers assessed varied with the exception of steroid hormone receptor status, which was incorporated in the majority of analyses (except [27,29]). p53 was included as a co-variable in 11 studies [21,26-28,31-34,37,39,40] and HER2 in three [21,26-28,31-34,37,39,40]. A monoclonal antibody to assess BCL2, either from Dako (Clone 124) [21,26-28,31-34,37,39,40], Novacastra [29] or Dakopatts [30,36,38,41] was used by most investigators. A polyclonal antibody was used in one study [35]. Cytoplasmic staining was scored using a dichotomous scoring system in all studies with a cut-off for positive status between 10% and 40%. Berardo *et al*. [34] applied both continuous and dichotomous system and showed that the former was associated with independent significance but not the latter.

**Figure 1:**
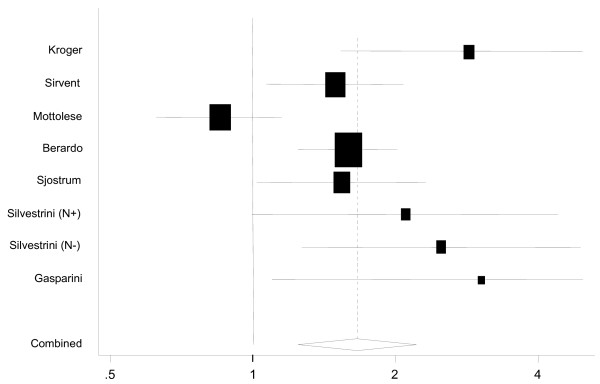
Funnel plot of the combined relative hazard from the random effects models for disease free survival in BCL2 negative cases compared to BCL2 positive cases in univariate analysis.

Eight studies including 2,285 patients reported the effect of BCL2 expression on DFS in analyses unadjusted for other risk factors (Figure 1, Table 1). The unadjusted HR estimates for DFS from these studies ranged from 0.85 – 3.03 and all but one of these was significant at a nominal *p *< 0.05. The pooled random effects estimate was 1.66 (95% CI = 1.25 – 2.22). However, there was evidence for significant heterogeneity amongst the studies (Q = 22.4, 7 degrees of freedom (df), *p *= 0.002), which might be expected given the difference in populations being studied and experimental methods used. Five studies [26,34,35,38,41] included node-positive disease only accounting for 1,659 cases. Notwithstanding that, the heterogeneity was largely due to the study reported by Mottolese *et al*. [32], which was the only study to report a better outcome for BCL2 negative tumours. After the exclusion of the latter, the pooled estimate of HR was 1.74 (95%CI = 1.46–2.07) with no significant heterogeneity (Q = 6.6, 6 df, *p *= 0.36). There was no evidence for publication bias (*p *= 0.14). Eight studies were excluded from the primary analysis because they were small or because the HR was not reported but could be estimated from the data presented [43-48,52,53]. The pooled HR for these studies was 2.11 (95%CI = 1.62 – 2.77). This is significantly different from the estimate for the other studies (*p *= 0.02) suggesting, as predicted, a substantial bias in the HR estimates for the smaller studies and those with less completely reported data. If, however, all studies were analysed together the pooled HR estimate was 1.59 (95%CI = 1.41 – 1.81).

**Table 1:** Results of meta-analysis of expression of Bcl-2 and outcome in Breast Cancer.

**Endpoint and meta-analysis model**	**Estimate of Relative Hazard**			**Homogeneity Test**	
		(95% CI)	*p*	Q (df)	p
**Unadjusted Disease-Free Survival **(8 studies, n = 2285)					
Fixed	1.5	(1.3–1.7)	<0.001	22.4 (7)	0.002
Random	1.7	(1.3–2.2)	<0.001		
Fixed *	1.7	(1.5–2.0)	<0.001	6.6 (6)	0.360
Random*	1.7	(1.5–2.1)	<0.001		
**Adjusted Disease-Free Survival **(11 studies, n = 2128)					
Fixed	1.5	(1.3–1.7)	<0.001	17.2 (10)	0.07
Random	1.6	(1.3–2.0)	<0.001		
**Unadjusted Overall Survival **(8 studies, n = 3910)					
Fixed	1.6	(1.4–1.7)	<0.001	17.5 (7)	0.015
Random	1.6	(1.4–2.0)	<0.001		
Fixed *	1.6	(1.5–1.8)	<0.001	10.0 (6)	0.126
Random*	1.7	(1.5–2.0)	<0.001		
**Adjusted Overall Survival **(9 studies, n = 3624)					
Fixed	1.3	(1.2–1.4)	<0.001	15.5 (8)	0.050
Random	1.4	(1.2–1.6)	<0.001		

* values after exclusion of the study by Motolesse *et al*. [31] (n = 157)

Eleven studies including 2,105 patients reported the effects of BCL2 on DFS adjusted for other prognostic factors [26,27,29,30,33,35-39,41] (Table 1). The adjusted HR estimates were generally close to one but ranged from 1.10 to 3.26. In five of these studies incorporating 950 cases, BCL2 was an independent predictor of DFS. The pooled random effects HR estimate for these studies was 1.58 (95%CI = 1.29–1.94) without evidence for significant heterogeneity between the studies (Q = 17.2, 10 df,*p *= 0.07) or publication bias (*p *= 0.78). Four of the studies that failed to show an independent association between BCL2 and DFS used the Dakopatts or a polyclonal antibody.

**Figure 2:**
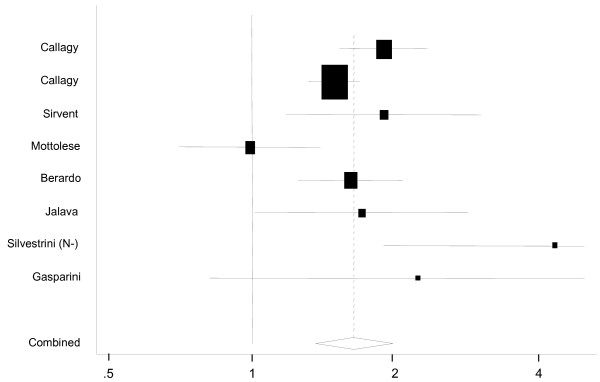
Funnel plot of the combined relative hazard from the random effects models for overall survival for BCL2 negative cases compared to BCL-2 positive cases in univariate analysis.

The effect of BCL2 expression on OS was evaluated in 12 studies in 11 reports (Table 1). HRs were unadjusted in three of these [32,34,40] and adjusted for other variables in four [28,36,37,39] and both unadjusted and adjusted HRs were given in four other reports [21,27,31,41]. The eight studies from seven reports [21,27,31,41] incorporating 3,910 cases where the expression of BCL2 was unadjusted for other variables produced hazard estimates ranging from 0.99–4.31 (Figure 2), of which 6 were statistically significant with a pooled estimate of risk of 1.64 (95%CI = 1.36–1.97). There was evidence of statistical heterogeneity (Q = 17.5, 7 df, *p *= 0.015) that again was virtually entirely due to the contribution of the report by Mottolese *et al*. After exclusion of this study there was little change in the HR (pooled estimate 1.73, 95%CI = 1.48–2.02) and no evidence for heterogeneity (Q = 9.98, 6 df, *p *= 0.13) or publication bias (*p *= 0.87).

Six studies were excluded from the primary analysis because they were small or because the HR was not reported but could be estimated from the data presented [44,48-52]. The pooled HR for these studies was 3.42 (95%CI = 2.41 – 4.86). This is significantly different from the estimate for the other studies (*p *< 0.001), again suggesting a substantial bias in the HR estimates for the smaller studies. If, however, all studies were analysed together the pooled HR estimate was 1.99 (95%CI = 1.62 – 2.45).

Nine series comprising 3,624 cases were used for the meta-analysis of the expression of BCL2 on OS adjusted for other parameters [21,27,28,31,36,37,39,41]. BCL2 was an independent predictor of outcome in four of these [21,36,39] totalling 2,190 cases. A narrow range of estimates was observed from 1.10 to 2.49 with a pooled estimate of 1.37 (95%CI = 1.19–1.58). There was borderline evidence of heterogeneity between the studies (Q = 15.48, 8 df, *p *= 0.05) and evidence of publication bias (*p *= 0.022).

## Discussion

The published data incorporating 5,892 cases strongly support the prognostic role of BCL2 as assessed by IHC in breast cancer showing that it is associated with both DFS and OS (pooled HR estimates of 1.66 and 1.64 respectively). These effects were slightly attenuated but still significant in multivariate analyses (adjusted HRs of 1.58 and 1.37 for DFS and OS respectively), showing that this effect is independent of lymph node status, tumour size and tumour grade as well as a range of other biological variables on multivariate analysis.

It is possible that the result of the meta-analysis could have been influenced by differences between the 18 studies. The finding of study heterogeneity in HR estimates is unsurprising and, even though the study by Mottolese *et al*. accounted for much of the heterogeneity for DFS, there were substantial differences between the other studies that could have influenced the result. Different cohorts of patients and treatment regimes were used. Five studies used unselected consecutive series [21,29,31,33,39] and these patients were managed according to standard treatment protocols. In all of these reports, BCL2 was an independent predictor of either DFS [29,33,39] or OS [21,31,39] on multivariate analyses. Treatments varied where cases were part of a clinical trial [21,26,32,35,37] although BCL2 was still an independent predictor of DFS and OS in the majority ([26,37] and [21,37] respectively). The cause of the heterogeneity from the study of Mottolese *et al*. is unclear. The series was part of a clinical trial that examined the influence of a number of patho-biological factors on response to adjuvant therapy. Patient characteristics, type of antibody and scoring system were similar to those used by others [21]. However, the authors showed an association between BCL2 positivity and adverse outcome, which is at variance with most other published reports and with all others included in the meta-analysis.

The types of antibodies employed and cut-offs used to define immunohistochemical positivity also varied between reports. The effect of each of these is difficult to assess but could have contributed to the observed heterogeneity. Clone 124 was the most commonly used and was used in the majority of studies where BCL2 was an independent predictor of DFS or OS [21,26-28,31,33,37,39,40]. In contrast, most reports that used the Dakopatts monoclonal antibody or a polyclonal antibody [30,35,36,41] failed to show a statistically significant association with DFS or OS, although the direction of effect was the same. There was little difference in the scoring systems between the studies although the different cut-off points varied from 10% to 40%. The inclusion of different combinations of variables in multivariate analyses could also have affected the results of a pooled estimate of the adjusted HRs. The purpose of the meta-analysis was to establish if the association of BCL2 with prognosis is independent of other confounders. The majority of studies included the most important variables in their analysis, which suggests that the pooled adjusted estimate of hazard is robust and supports BCL2 as an independent risk factor. In addition, the similarity between the HRs observed for BCL2 in both univariate and multivariate analyses, where both types of analyses were performed also attest to its prognostic role.

The effect of bias on the meta-analysis should also be considered. Seventeen eligible reports provided data suitable for the meta-analysis. Thirty six eligible studies were excluded, including studies using small series and those that did not provide minimum data for the pooled analysis. The exclusion of small studies may have minimised the effect of publication bias – the non-publication of studies with null results – by not including reports of small series that are more likely to be published if they show a positive result. This is supported by our findings that the HRs reported by smaller studies were systematically larger than those reported by the larger studies. The inclusion of only large studies and those that meet minimum quality criteria in the meta-analysis maximised the chance of the pooled estimate of HR representing the true HR. Whether we have been able to retrieve all relevant reports in the literature is unclear. In particular, it is possible that our search strategy will have missed studies evaluating BCL2 as one of multiple markers, but where no association with outcome was detected. This is perhaps most likely to occur in studies using TMAs. However, if large negative studies had been missed, a substantial degree of publication bias (perhaps a misnomer here as the data may be published and not retrieved) would have been detected, whereas we found little evidence for this. It seems unlikely that missing data bias either through non-publication, or through failure to identify and retrieve the data, would have led to a substantial overestimation of the meta-analysis HR estimates.

The mechanisms through which BCL2 might exert its protective effect in breast cancer are unclear. Whether it is consequent upon its role in apoptosis or whether non-apoptotic functions are involved is unknown. The anti-apoptotic role of BCL2 is well characterised but its function in cell cycle control has received less attention. The latter is well supported by cell line studies that show BCL2 expression delays G1 progression and G1-S transition by prolonging G0 and is capable of growth inhibitory effects analogous to those of p53 [24,57]. It is postulated that the dominance of one of these functions over another may depend on the cell type and physiology and that the anti-proliferative effect translates into a tumour suppressor role in solid epithelial tumours including breast cancer. Furthermore, it remains to be established if other proteins *e.g*. Wnt11 [58] or bcl homologues potentiate the tumour suppressor role of BCL2 in breast cancer. At least 20 bcl proteins are known that may act in a synergistic manner, analogous to the co-operative effect of MYC and BCL2 in lymphomagenesis.

The therapeutic implication of our findings and how BCL2 might improve prognostication and/or selection of patients for treatment remain to be determined. The data presented here shows that BCL2 has the potential to improve patient stratification and guide patient management. Currently, patients are stratified into different treatment categories based primarily on nodal status, tumour size, tumour grade, receptor status, patient age and, to a lesser extent, tumour type. A range of guidelines *e.g*. St. Gallen, National Institutes of Health Consensus Development Panel, National Comprehensive Cancer Network (NCCN) Clinical Practice Guidelines and prognostic systems *e.g*. NPI, ADJUVANT! are used internationally, each of which combines these features in different ways to assign patients to risk categories. Recommendations for systemic treatment are made by some, for example, the NCCN recommends adjuvant chemotherapy for tumours > 1 cm in combination, with adjuvant hormonal and traztuzumab depending on receptor status, but not for small (≤ 5 mm), node-negative invasive tumours or for node-negative grade 1 tumours between 6 mm and 1 cm. In the UK, the NPI is used widely to inform decisions on adjuvant therapy [59,60], however, there is no agreement on cut-off values. The prognostic value of the NPI is supported from validation studies but no new prognostic factors have been shown that add substantially to its use. Taken in conjunction with previous data, our results suggest that BCL2 could improve the stratification of patients by the NPI and could separate both the moderate and the poor prognostic groups each into two prognostic categories. We showed previously that the prognostic effect of BCL2 status is maximal in the first five years after a diagnosis of breast cancer and wanes thereafter, suggesting that its utility as a diagnostic adjunct may be limited to this period. Even if BCL2 were to be used in clinical decision making, the most appropriate cut-off used to assign positivity is uncertain. Our previous report [21] indicated that a dichotomous scoring system using a 10% cut-off provided the most parsimonious fit for predicting overall survival using a cox regression analysis, but published data are insufficient to replicate this.

The number of studies included in the meta-analysis with different treatment endpoints was too small to perform a meaningful analysis of its predictive role. However, there is an emerging consensus that BCL2 plays a key role in determining response to endocrine therapy and chemotherapy [61]. BCL2 is an oestrogen responsive gene [58,62] and many clinical studies have shown an association with favourable response to endocrine therapy [40,61,63]. BCL2 is a component of the 21-gene signature used to predict recurrence in tamoxifen-treated ER-positive node-negative breast cancer and its role is being evaluated prospectively in the TAILORx trial [14].

## Conclusion

In summary, we have shown that BCL2 is an independent prognostic marker in two large series of breast cancer [21] and, here, provide an estimate of the average size of this association with outcome in a meta-analysis on 16 other series totalling over 5,000 patients. Using this approach, we have demonstrated the prognostic power of a single marker in breast cancer. Large studies, both observational cohorts and clinical trials, are now urgently needed to test whether BCL2 and multiple other markers can provide prognostic information in addition to currently used standards and also to establish if BCL2 has clinical utility.

## List of abbreviations

CI: confidence interval; df: degrees of freedom; DFS: disease free survival; EC: epirubicin and cyclophosphamide; G-CSF: granulocyte colony stimulating factor; HER2: Human epidermal growth factor receptor; HR: hazard ratio; IHC: immunohistochemistry; NCCN: National Comprehensive Cancer Network; NPI: Nottingham Prognostic Index; OS: overall survival; TAILORx: Trial Assigning Individualized Options for Treatment (Rx); TMA: tissue micro-array; TNM: tumour, nodal status, metastasis; UICC: Union Internationale Contre le Cancer.

## Competing interests

The authors declare that they have no competing interests.

## Authors' contributions

MJW performed the search of pubmed and organised the data. GMC organised the data, drafted and finalised the manuscript. PDPP performed the meta-analysis. PDPP and CC both contributed to writing and finalising of the manuscript. All authors read and approved the final manuscript.

## Pre-publication history

The pre-publication history for this paper can be accessed here:



## Supplementary Material

Additional file 1 Table of 36 studies excluded from the meta-analysis. table summarising the 36 studies excluded from the meta-analysis on the basis of small study size and the provision of insufficient data for the pooled analysis. Click here for file

Additional file 2 References for 36 studies excluded from meta-analysis. References for the 36 studies summarised in additional file 1 that were excluded from meta-analysis. Click here for file

Additional file 3 Reports examining the association between expression of BCL2 and DFS and/or OS that include over 100 cases of breast cancer. table summarising the 17 reports included for the meta-analysis. Click here for file

Additional file 4 Results of univariate and multivariate analyses for OS and DFS for 17 studies included in the meta-analysis. table summarising the results of univariate and multivariate analysis in 17 reports included for the meta-analysis. Click here for file
