# Supplementary material for: Meta-analysis confirms BCL2 is an independent prognostic marker in breast cancer
Source: BMC Cancer. 2008 May 29;8:153. doi: 10.1186/1471-2407-8-153 (PMC2430210; doi:10.1186/1471-2407-8-153)
Supplement: Additional file 2 — References for 36 studies excluded from meta-analysis. References for the 36 studies summarised in additional file 1 that were excluded from meta-analysis. [file 1471-2407-8-153-S2.doc]

**References for 36 studies excluded from meta-analysis summarised in additional file 1**

1. Krajewski S, Blomqvist C, Franssila K, Krajewska M, Wasenius VM, Niskanen E, Nordling S, Reed JC: **Reduced expression of proapoptotic gene BAX is associated with poor response rates to combination chemotherapy and shorter survival in women with metastatic breast adenocarcinoma**. *Cancer Res* 1995, **55**(19):4471-4478.

2. Lipponen P, Pietilainen T, Kosma VM, Aaltomaa S, Eskelinen M, Syrjanen K: **Apoptosis suppressing protein bcl-2 is expressed in well-differentiated breast carcinomas with favourable prognosis**. *J Pathol* 1995, **177**(1):49-55.

3. Al-Moundhri M, Nirmala V, Al-Mawaly K, Ganguly S, Burney I, Rizvi A, Grant C: **Significance of p53, Bcl-2, and HER-2/neu protein expression in Omani Arab females with breast cancer**. *Pathol Oncol Res* 2003, **9**(4):226-231.

4. Arun B, Kilic G, Yen C, Foster B, Yardley D, Gaynor R, Ashfaq R: **Correlation of Bcl-2 and p53 expression in primary breast tumors and corresponding metastatic lymph nodes**. *Cancer* 2003, **98**(12):2554-2559.

5. Beenken SW, Grizzle WE, Crowe DR, Conner MG, Weiss HL, Sellers MT, Krontiras H, Urist MM, Bland KI: **Molecular biomarkers for breast cancer prognosis: coexpression of c-erbB-2 and p53**. *Ann Surg* 2001, **233**(5):630-638.

6. Bhatavdekar JM, Patel DD, Shah NG, Vora HH, Suthar TP, Chikhlikar PR, Ghosh N, Trivedi TI: **Prognostic significance of immunohistochemically localized biomarkers in stage II and stage III breast cancer: a multivariate analysis**. *Ann Surg Oncol* 2000, **7**(4):305-311.

7. Bukholm IR, Bukholm G, Nesland JM: **Reduced expression of both Bax and Bcl-2 is independently associated with lymph node metastasis in human breast carcinomas**. *Apmis* 2002, **110**(3):214-220.

8. Dimitrakakis C, Konstadoulakis M, Messaris E, Kymionis G, Karayannis M, Panoussopoulos D, Michalas S, Androulakis G: **Molecular markers in breast cancer: can we use c-erbB-2, p53, bcl-2 and bax gene expression as prognostic factors?** *Breast* 2002, **11**(4):279-285.

9. Giatromanolaki A, Koukourakis MI, Kakolyris S, Mavroudis D, Kouroussis C, Mavroudi C, Perraki M, Sivridis E, Georgoulias V: **Assessment of highly angiogenic and disseminated in the peripheral blood disease in breast cancer patients predicts for resistance to adjuvant chemotherapy and early relapse**. *Int J Cancer* 2004, **108**(4):620-627.

10. Lee HD, Koo JY, Jung WH: **Correlations of bcl-2 expression with clinicopathological features in breast cancer**. *Yonsei Med J* 1997, **38**(4):206-211.

11. Lee WY, Su WC, Lin PW, Guo HR, Chang TW, Chen HH: **Expression of S100A4 and Met: potential predictors for metastasis and survival in early-stage breast cancer**. *Oncology* 2004, **66**(6):429-438.

12. Linjawi A, Kontogiannea M, Halwani F, Edwardes M, Meterissian S: **Prognostic significance of p53, bcl-2, and Bax expression in early breast cancer**. *J Am Coll Surg* 2004, **198**(1):83-90.

13. Schiller AB, Clark WS, Cotsonis G, Lawson D, DeRose PB, Cohen C: **Image cytometric bcl-2:bax and bcl-2:bcl-x ratios in invasive breast carcinoma: correlation with prognosis**. *Cytometry* 2002, **50**(4):203-209.

14. Sjostrom J, Blomqvist C, von Boguslawski K, Bengtsson NO, Mjaaland I, Malmstrom P, Ostenstadt B, Wist E, Valvere V, Takayama S *et al*: **The predictive value of bcl-2, bax, bcl-xL, bag-1, fas, and fasL for chemotherapy response in advanced breast cancer**. *Clin Cancer Res* 2002, **8**(3):811-816.

15. Vakkala M, Lahteenmaki K, Raunio H, Paakko P, Soini Y: **Apoptosis during breast carcinoma progression**. *Clin Cancer Res* 1999, **5**(2):319-324.

16. Wu J, Shao ZM, Shen ZZ, Lu JS, Han QX, Fontana JA, Barsky SH: **Significance of Apoptosis and Apoptotic-Related Proteins, Bcl-2, and Bax in Primary Breast Cancer**. *Breast J* 2000, **6**(1):44-52.

17. Bottini A, Berruti A, Bersiga A, Brizzi MP, Bruzzi P, Aguggini S, Brunelli A, Bolsi G, Allevi G, Generali D *et al*: **Relationship between tumour shrinkage and reduction in Ki67 expression after primary chemotherapy in human breast cancer**. *Br J Cancer* 2001, **85**(8):1106-1112.

18. Joensuu H, Pylkkanen L, Toikkanen S: **Bcl-2 protein expression and long-term survival in breast cancer**. *Am J Pathol* 1994, **145**(5):1191-1198.

19. Barbareschi M, Caffo O, Veronese S, Leek RD, Fina P, Fox S, Bonzanini M, Girlando S, Morelli L, Eccher C *et al*: **Bcl-2 and p53 expression in node-negative breast carcinoma: a study with long-term follow-up**. *Hum Pathol* 1996, **27**(11):1149-1155.

20. Nakopoulou L, Giannopoulou I, Stefanaki K, Panayotopoulou E, Tsirmpa I, Alexandrou P, Mavrommatis J, Katsarou S, Davaris P: **Enhanced mRNA expression of tissue inhibitor of metalloproteinase-1 (TIMP-1) in breast carcinomas is correlated with adverse prognosis**. *J Pathol* 2002, **197**(3):307-313.

21. van Slooten HJ, Clahsen PC, van Dierendonck JH, Duval C, Pallud C, Mandard AM, Delobelle-Deroide A, van de Velde CJ, van de Vijver MJ: **Expression of Bcl-2 in node-negative breast cancer is associated with various prognostic factors, but does not predict response to one course of perioperative chemotherapy**. *Br J Cancer* 1996, **74**(1):78-85.

22. Krajewski S, Thor AD, Edgerton SM, Moore DH, 2nd, Krajewska M, Reed JC: **Analysis of Bax and Bcl-2 expression in p53-immunopositive breast cancers**. *Clin Cancer Res* 1997, **3**(2):199-208.

23. Charpin C, Garcia S, Bonnier P, Martini F, Andrac L, Horschowski N, Lavaut MN, Allasia C: **Bcl-2 automated quantitative immunocytochemical assays in breast carcinomas: correlation with 10-year follow-up**. *J Clin Oncol* 1998, **16**(6):2025-2031.

24. Veronese S, Mauri FA, Caffo O, Scaioli M, Aldovini D, Perrone G, Galligioni E, Doglioni C, Dalla Palma P, Barbareschi M: **Bax immunohistochemical expression in breast carcinoma: a study with long term follow-up**. *Int J Cancer* 1998, **79**(1):13-18.

25. Cardoso F, Paesmans M, Larsimont D, Durbecq V, Bernard-Marty C, Rouas G, Dolci S, Sotiriou C, Piccart MJ, Di Leo A: **Potential predictive value of Bcl-2 for response to tamoxifen in the adjuvant setting of node-positive breast cancer**. *Clin Breast Cancer* 2004, **5**(5):364-369.

26. Daidone MG, Veneroni S, Benini E, Tomasic G, Coradini D, Mastore M, Brambilla C, Ferrari L, Silvestrini R: **Biological markers as indicators of response to primary and adjuvant chemotherapy in breast cancer**. *Int J Cancer* 1999, **84**(6):580-586.

27. Koukourakis MI, Giatromanolaki A, Galazios G, Sivridis E: **Molecular analysis of local relapse in high-risk breast cancer patients: can radiotherapy fractionation and time factors make a difference?** *Br J Cancer* 2003, **88**(5):711-717.

28. McCallum M, Baker C, Gillespie K, Cohen B, Stewart H, Leonard R, Cameron D, Leake R, Paxton J, Robertson A *et al*: **A prognostic index for operable, node-negative breast cancer**. *Br J Cancer* 2004, **90**(10):1933-1941.

29. Kapranos N, Karaiosifidi H, Valavanis C, Kouri E, Vasilaros S: **Prognostic significance of apoptosis related proteins Bcl-2 and Bax in node-negative breast cancer patients**. *Anticancer Res* 1997, **17**(4A):2499-2505.

30. O'Driscoll L, Cronin D, Kennedy SM, Purcell R, Linehan R, Glynn S, Larkin A, Scanlon K, McDermott EW, Hill AD *et al*: **Expression and prognostic relevance of Mcl-1 in breast cancer**. *Anticancer Res* 2004, **24**(2A):473-482.

31. Abdulkader I, Sanchez L, Cameselle-Teijeiro J, Gude F, Chavez JE, Lopez-Lopez R, Forteza J, Fraga M: **Cell-cycle-associated markers and clinical outcome in human epithelial cancers: a tissue microarray study**. *Oncol Rep* 2005, **14**(6):1527-1531.

32. Choi DH, Kim S, Rimm DL, Carter D, Haffty BG: **Immunohistochemical biomarkers in patients with early-onset breast carcinoma by tissue microarray**. *Cancer J* 2005, **11**(5):404-411.

33. Hlupic L, Jakic-Razumovic J, Bozikov J, Coric M, Belev B, Vrbanec D: **Prognostic value of different factors in breast carcinoma**. *Tumori* 2004, **90**(1):112-119.

34. Schneeweiss A, Katretchko J, Sinn HP, Unnebrink K, Rudlowski C, Geberth M, Beldermann F, Bastert G, Strittmatter HJ: **Only grading has independent impact on breast cancer survival after adjustment for pathological response to preoperative chemotherapy**. *Anticancer Drugs* 2004, **15**(2):127-135.

35. Elledge RM, Green S, Ciocca D, Pugh R, Allred DC, Clark GM, Hill J, Ravdin P, O'Sullivan J, Martino S *et al*: **HER-2 expression and response to tamoxifen in estrogen receptor-positive breast cancer: a Southwest Oncology Group Study**. *Clin Cancer Res* 1998, **4**(1):7-12.

36. Bankfalvi A, Tory K, Kemper M, Breukelmann D, Cubick C, Poremba C, Fuzesi L, Lelle RJ, Bocker W: **Clinical relevance of immunohistochemical expression of p53-targeted gene products mdm-2, p21 and bcl-2 in breast carcinoma**. *Pathol Res Pract* 2000, **196**(7):489-501.
