# Supplementary material for: Meta-analysis confirms BCL2 is an independent prognostic marker in breast cancer
Source: BMC Cancer. 2008 May 29;8:153. doi: 10.1186/1471-2407-8-153 (PMC2430210; doi:10.1186/1471-2407-8-153)
Supplement: Additional file 3 — Reports examining the association between expression of BCL2 and DFS and/or OS that include over 100 cases of breast cancer. table summarising the 17 reports included for the meta-analysis. [file 1471-2407-8-153-S3.doc]

Reports examining the association between expression of BCL2 and DFS and/or OS that include over 100 cases of breast cancer

| **Author** | **n^a^** | **Type of series** | **Median**  **follow-up (range)** ^∫^ | **Antibody** | **Bcl-2 scoring system (cutoff)** | **Variables included in analysis** | **Comment** |
| --- | --- | --- | --- | --- | --- | --- | --- |
| **Callagy, 2006 [21]^₣^** | 728 | Clinical trial | 104.4 (4.8-473) | M^†^ | D (10%) | Nodal status, tumour size, grade, ER, PR, BCL2, cyclin E, p53, MIB1, mcm-2, CK5/6, CK17, CK 8/18, HER2, p-27, cMyc | Cases selected from a series of 2,475 women with stage I - III breast cancer who participated in five different British Columbia Cancer Agency clinical trials between 1970 and 1990. |
| **Callagy, 2006[21]^₣^** | 983 | Unselected | 648 (216-840)* | M^†^ | D (10%) | Nodal status, tumour size, grade, ER, PR, BCL2 | A consecutive cases of primary operable breast carcinoma patients presenting from 1986-1998 and entered into the Nottingham Tenovus Primary Breast Carcinoma Series. |
| **Kroger, 2006 [25]** | 157 | Node-positive | 45.6 | M^†^ | D (see comment) | Tumour size, grade, therapy (surgery, chemotherapy, radiotherapy, endocrine), menopausal status, ER, PR, HER2, BCL2, Ki-67, p16, p53, maspin | Cases selected from the German Adjuvant Breast Cancer prospective randomised trial of high dose chemotherapy in women with > 10 positive nodes. BCL2 expression was scored as negative, weak positive, strong positive based on an index using percentage of positive cells and intensity of staining. |
| **Sirvent, 2004 [26]** | 186 | Consecutive pT1-2 IDC | 54.6 (1-138) | M^†^ | D (10%) | Nodal status, tumour size, grade, tumour necrosis, apoptotic index, BCL2, Bag-1, Bax, p53 |  |
| **Chang, 2003 [27]** | 346 | Node positive | 346 | M^†^ | D (see comment) | Nodal status, tumour size, therapy, age, ER, PR, BCL2, SPF, ploidy, p53, Ki-67, HER2 | Endpoint for HR is survival from first recurrence. BCL2 expression was scored using an index (0-8) of percentage of positive cells and intensity (cut-off ≥6). |
| **Yang, 2003 [28]** | 147 | Unselected | 53 (2-106) | M^§^ | D (10%) | Nodal status, tumour size, grade, BCL2 |  |
| **Kymionis, 2001 [29]** | 130 | Node negative | 66 (3-109) | M^‡^ | D (10%) | Tumour size, grade, ER, PR, BCL2, bax |  |
| **Jalava, 2000 [30]** | 414 | Unselected |  | M^†^ | D (>control) | Nodal status, tumour size, tumour type, grade, ER, BCL2, MPI, SMI, Ki-67, DNA index | Patients enrolled between1988-1991 and clinical data collected in 1996. Length of follow-up not provided. |
| **Mottolese, 2000 [31]** | 157 | Clinical trial | 60 | M^†^ | D (20%) | Nodal status, tumour size, grade, type, menopausal status, ER, PR, HER2, p53, BCL2, Ki-67 | Cases selected from clinical trial of adjuvant chemotherapy. Patients were high-risk according to St Gallen criteria. Treatment between 1991 and 1993. Patients assigned randomly to treatment with Lonidamide and/or G-CSF and EC chemotherapy. |
| **Le, 1999 [32]** | 175 | Unselected | 114* | M^†^ | D (30%) | Nodal status, tumour size, grade, UICC stage, ER, PR, BCL2, p53, c-Myc |  |
| **Berardo, 1998 [33]** | 979 | Node Positive, M0 | 61 | M^†^ | D and C (see comment) | Nodal status, tumour size, age, ER, PR, p53, SPF, percentage of apoptotic cells, DNA ploidy | BCL2 was scored using an index of percentage of positive cells and intensity (cut-off ≥6). |
| **Sjostrum, 1998 [34]** | 103 | Clinical trial | 34.7 (22.4-66.1) | P | D (10%) | Tumour grade, ER, PR, HER2, SPF, DNA ploidy, p53, bax | Patients with metastatic disease enrolled on a randomised trial between 1987 and 1991 and received 5-fluorouracil, EC in four weekly doses or in an every-4-week dose. Length of follow-up is for 11 surviving patients. All other patients were followed until death. |
| **Zhang, 1997 [35]** | 228 | Node positive, M0 | 132 (13.2-180)* | M^‡^ | D (10%) | Nodal status, tumour size, grade, tumour type, TNM stage, age, menopausal status, ER, PR, HER2, BCL2, p53 |  |
| **Elledge, 1997**  **[36]** | 205 | Clinical trial |  | M^†^ | D (10%) | Menopausal status, age, visceral disease, metastasis at presentation, disease free interval, adjuvant therapy, ER, PR, BCL2, p53 |  |
| **Silvestrini, 1996 [37]** | 240 | postmenopausal, node-positive, ER positive | 60 | M^‡^ | D (40%) | Nodal status, tumour size, ER, PR, TLI, p53 |  |
| **Hellemans, 1995 [38]** | 251 | Unselected | 91 (24-186) | M^†^ | D (10%) | Nodal status, tumour size, grade, menopausal status, metastasis, ER, PR, BCL2 |  |
| **Gasparini, 1995 [39]** | 180 | Consecutive T1-T3a, N1-2, M0, ER negative | 72* | M^†^ | D (25%) | Nodal status, tumour size, grade, tumour type, age, menopausal status, therapy, ER, PR, BCL2, p53 |  |
| **Silvestrini, 1994 [40]** | 283 | *Node-negative | 72 (6-140) | M^‡^ | D (30%) | Tumour size, TLI, ER, BCL2, p53 |  |

a Number of events for each study [reference] where given: [21a] 476 deaths, 512 events; [21b] 187 deaths; [25] 148 events; [28] 28 events; [29] 36 deaths and 43 events; [36] 179 events; [37] 142 events; [39] 58 deaths and 76 events.

∫ follow-up given in months in all cases;

₣, two series were examined in this report;

* mean follow-up in months;

† Dako, clone 124; ‡ Dakopatts; §, Novacastra.

Abbreviations: CK, cytokeratin; EC, epirubicin and cyclophosphamide; G-CSF, granulocyte colony stimulating factor; M, monoclonal; MPI, morphometric index; P, polyclonal; SMI, standardised mitotic index; SPF, S-phase fraction; TLI, thymidine labelling index; TNM, tumour, nodal status, metastasis; UICC, Union Internationale Contre le Cancer.
