# Supplementary material for: Meta-analysis confirms BCL2 is an independent prognostic marker in breast cancer
Source: BMC Cancer. 2008 May 29;8:153. doi: 10.1186/1471-2407-8-153 (PMC2430210; doi:10.1186/1471-2407-8-153)
Supplement: Additional file 4 — Results of univariate and multivariate analyses for OS and DFS for 17 studies included in the meta-analysis. table summarising the results of univariate and multivariate analysis in 17 reports included for the meta-analysis. [file 1471-2407-8-153-S4.doc]

Results of univariate and multi-variate analysis for OS and DFS for 17 studies included in the meta-analysis

| **Author** | **Baseline reference cate gory ^₣^** | **Univariate analysis** | | **Variables in multivariate analysis** | **Multivariate analysis** | | **Comments** |
| --- | --- | --- | --- | --- | --- | --- | --- |
|  |  | **Relative Hazard (95% CI) for BCL2** | |  | **Relative Hazard (95% CI) for BCL2** | |  |
|  |  | **RFS/DFS** | **OS** |  | **RFS** | **OS** |  |
| **Callagy,**  **2006a [21]** | N^†^ |  | 0.53 (0.43-0.66) | ER, PR, HER2, BCL2, Cyclin E, p53, MIB-1, CK5/6, NPI |  | 0.62 (0.44-0.87) | Complete data for multivariate analysis available for 403 cases |
| **Callagy,**  **2006b [21]** | N^†^ |  | 0.67 (0.59-0.76) | ER, PR, BCL2, NPI |  | 0.83 (0.71 -0.96) | Complete data for multivariate analysis available for 928 cases |
| **Kroger,**  **2006 [25]** | N^†^ | 0.35 (0.19-0.66)  0.36 (0.17-0.76) |  | Tumour size, treatment, ER, HER2, p53, BCL2 | 0.35 (0.19-0.64) |  | In univariate analysis, HRs were given both for weak and for strong positive *versus* negative. The former was used for the meta-analysis. Both positive categories were combined for multivariate analysis. |
| **Sirvent,**  **2004 [26]** | N^§^ | 0.67 (0.46-0.9) | 0.53 (0.33-0.85­) | Nodal status, tumour size, tumour necrosis, p53, BCL-2, BAG-1, Bax | 0.88 (0.7-0.9) | 0.84 (0.72-0.96) |  |
| **Chang,**  **2003 [27]** | P^†^ |  |  | Age at diagnosis, visceral metastasis, shorter DFI, PR negativity; increased SPF; BCL-2 negativity |  | 1.23 (1.00-1.67) |  |
| **Yang,**  **2003 [28]** | P^‡^ |  |  | Nodal status, tumour size, grade, BCL2 | 3.26 (1.17-9.09) |  |  |
| **Kymionis, 2001 [29]** | N^§^ |  |  | Tumour size, grade, post-operative treatment, ER, PR, bas , BCL2, Bax | 0.72 (0.30-1.73) |  |  |
| **Jalava,**  **2000 [30]** | P^§^ |  | 1.7 (1.00-2.8­) | Nodal status, tumour size, grade, SMI, BCL2 |  | 1.1 (0.6-1.8) |  |
| **Mottolese, 2000 [31]** | N^§^ | 1.17 (0.86-1.58) | 1.01 (0.71-1.41) | Not indicated. Metastatic nodes and p53 in final model. |  |  |  |
| **G.Le,**  **1999 [32]** | N^§^ |  |  | Nodal status, UICC tumour size, grade, c-myc (mRNA), p53, steroid hormones, BCL2 | 0.4 (0.2-0.8­) |  |  |
| **Berardo,**  **1998 [33]** | N^§^ | 0.63 | 0.62 | Nodal status, tumour size, age, ER, PR, p53, ploidy, SPF | 0.96 |  | The BCL2 score was used as a continuous variable for multivariate analysis and could not be included in the meta-analysis. |
| **Sjostrum, 1998 [34]** | Not indicated^‡^ | 0.65 (0.43-0.98) |  | Tumour grade, PR, BCL2 , Bax | 0.91 (0.54- 1.51) |  |  |
| **Zhang,**  **1997 [35]** | P^†^ |  |  | Nodal status, tumour size, grade, age, menopausal status, TNM, HER2, p53, BCL2 | 1.26 (0.66-2.41) | 2.29 (1.27-3.39) |  |
| **Elledge,**  **1997 [36]** | N^†^ |  |  | Menopausal status, visceral metastasis, disease free interval, metastasis at presentation, ER, PR, adjuvant therapy, p53, BCL2 | 0.66 (0.5-0.89) | 0.75 (0.55 - 1.03) |  |
| **Silvestrini, 1996 [37]** | P^‡^ | 2.1 (1-4.45) |  | Nodal status, tumour size, ER, PR, p53, BCL2, TLI | 1.6 (0.9-3.0) |  |  |
| **Hellemans, 1995 [38]** | P^†^ |  |  | Nodal status, tumour size, grade, ER, BCL2 | 2.08 (1.25-3.45) | 2.49 (1.43 -4.33) |  |
| **Gasparini, 1995 [39]** | P ^‡^ | 3.03 (1.20-9.09) | 2.22 (0.84-6.25) | Nodal status, tumour size, ER, PR, p53, BCL2 |  |  | Multivariate analysis was performed for a subset of the larger series and so was excluded from the meta-analysis |
| **Silvestrini, 1994 [40]** | P^‡^ | 2.50 (1.14-4.42) | 4.31 (1.88-9.80) | Tumour size, ER, BCL2, p53, TLI | 1.53 (0.81-2.91) | 1.80 (0.79-4.09) |  |

₣ Authors used different measures to reflect risk as follows: †hazard ratio; § relative risk; ‡ odds ratio.

Note: Blank cells indicate that data not provided in report.

Abbreviations: CK, cytokeratin; SMI, standardised mitotic index; TLI, thymidine labelling index; TNM, tumour, nodal status and metastasis; UICC, Union Internationale Contre le Cancer.
